# Macrophages in Tumor Microenvironments and the Progression of Tumors

**DOI:** 10.1155/2012/948098

**Published:** 2012-06-19

**Authors:** Ning-Bo Hao, Mu-Han Lü, Ya-Han Fan, Ya-Ling Cao, Zhi-Ren Zhang, Shi-Ming Yang

**Affiliations:** ^1^Department of Gastroenterology, Xinqiao Hospital, Third Military Medical University, Chongqing 400037, China; ^2^Institute of Immunology, Third Military Medical University, Chongqing 400038, China; ^3^Biomedical Analysis Center, Third Military Medical University, Chongqing 40038, China; ^4^Chongqing Key Laboratory for Diseases Proteomics, Southwest Hospital, Third Military Medical University, Chongqing 400038, China

## Abstract

Macrophages are widely distributed innate immune cells that play indispensable roles in the innate and adaptive immune response to pathogens and in-tissue homeostasis. Macrophages can be activated by a variety of stimuli and polarized to functionally different phenotypes. Two distinct subsets of macrophages have been proposed, including classically activated (M1) and alternatively activated (M2) macrophages. M1 macrophages express a series of proinflammatory cytokines, chemokines, and effector molecules, such as IL-12, IL-23, TNF-**α**, iNOS and MHCI/II. In contrast, M2 macrophages express a wide array of anti-inflammatory molecules, such as IL-10, TGF-**β**, and arginase1. In most tumors, the infiltrated macrophages are considered to be of the M2 phenotype, which provides an immunosuppressive microenvironment for tumor growth. Furthermore, tumor-associated macrophages secrete many cytokines, chemokines, and proteases, which promote tumor angiogenesis, growth, metastasis, and immunosuppression. Recently, it was also found that tumor-associated macrophages interact with cancer stem cells. This interaction leads to tumorigenesis, metastasis, and drug resistance. So mediating macrophage to resist tumors is considered to be potential therapy.

## 1. Introduction

Macrophages were initially described by Elie Metchnikoff, who won the Nobel prize in 1905 because of his identification of phagocytes and his phagocytosis theory [1]. Since then, much progress has been made in revealing the mechanisms underlying macrophage activation and roles that macrophages play in our bodies. Today, it is well established that macrophages are important innate immune cells with essential roles in the primary response to pathogens, normal tissue homeostasis, presentation of foreign and self-antigens following infection or injury, resolution of inflammation, and wound healing. 

Macrophages exist in almost all tissues and play important roles in the maintenance of tissue homeostasis. In mature adults, macrophages differentiate from peripheral blood monocytes, which develop from common myeloid progenitor cells. These cells are identified as granulocyte/macrophage colony-forming units (GM-CFUs) in the bone marrow. In response to a macrophage colony-forming factor, GM-CFUs sequentially give rise to macrophage colony-forming units (M-CFUs), monoblasts, and pro-monocytes. Subsequently, they move into the peripheral blood and differentiate into monocytes. Finally, the monocytes migrate into different tissues and replenish the populations of long-lived tissue-specific macrophages, such as alveolar macrophages and kupffer cells [2, 3]. However, not all tissue macrophages are differentiated from monocytes. It has been reported that Langerhans cells in the skin and microglial cells in the brain, which are tissue-resident macrophage populations that are radiation resistant, seem to be maintained through local proliferation, and recent studies indicate that these cells initially develop from M-CFU in the yolk sac of the developing embryo [4]. 

**Figure 1:**
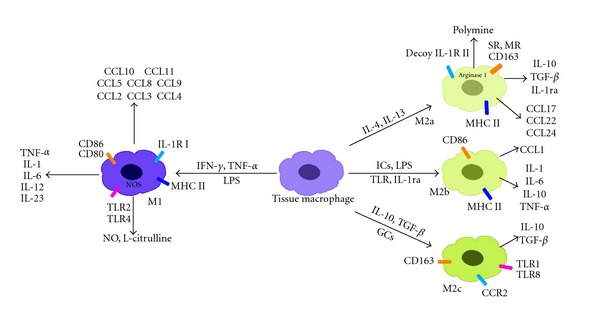
Classically and alternatively activated macrophages. Classically polarized macrophages are activated by LPS, IFN-*γ*, or TNF-*α*. Alternatively polarized macrophages can be further divided into M2a, M2b, and M2c macrophages. IL-4 and IL-13 always activate macrophages to be M2a macrophages. The main difference between M1 and M2a macrophages is in their metabolism of L-arginine. In M1 macrophages, L-arginine is metabolized into L-citrulline and NO by NOS2, while in M2a macrophages, it is metabolized into polyamine and urea by arginase 1. M2b macrophages are activated by immune complexes, TLRs, or IL-1ra. Finally, M2c macrophages are polarized by IL-10. All of the phenotypes express a series of different cytokines, chemokines, and receptors.

Macrophages, like other immune effector cells, can have multiple subtypes and take on various phenotypes depending on the microenvironment. By analogy to the Th1/Th2 classification, two distinct states of polarized activation for macrophages have been proposed: the classically activated (M1) macrophage and the alternatively activated (M2) macrophage subsets [5]. M1 macrophages arise following stimulation with the Th1 cytokine interferon-*γ* (IFN-*γ*) alone or in concert with bacterial moieties, such as lipopolysaccharide (LPS) or cytokines (e.g., tumor necrosis factor-*α* (TNF-*α*)) [6] (Figure 1). In contrast, M2 macrophages are polarized by distinct stimuli and can be further subdivided into M2a, M2b, and M2c macrophages. M2a macrophages are stimulated by the Th2 cytokines IL-4 or IL-13, and M2b macrophages are induced by immune complexes (ICs), LPS, TLRs, or the IL-1 receptor antagonist (IL-1ra). Finally, M2c macrophages are induced by IL-10, transforming growth factor-*β* (TGF-*β*), or glucocorticoids (GCs) [7] (Figure 1). M1macrophagessecretehighlevelsofpro-inflammatorycytokines (e.g., TNF-*α*, IL-1, IL-6, IL-12,andIL-23) and increase their concentrations of superoxide anions, oxygen radicals, and nitrogen radicals [8, 9]. Most ofthese agents can increasetheirkillingactivities. Furthermore, M1 macrophages can express high levels of MHC I and class II antigens and secrete complement factors that facilitate complement-mediated phagocytosis [10]. M1 macrophages can also secrete high levels of inducible nitric oxide synthase (iNOS; NOS2)to promote arginine metabolized into nitric oxide and citrulline [11]. Conversely, M2 macrophages always express the scavenger receptor (SR), the mannose receptor (MR), and IL-10, which lead M2 macrophages to mainly participate in parasite clearance, tissue remodeling, immune modulation, and tumor progression [9]. In this paper, we will discuss the characteristics of differentially polarized macrophages and explore the role of tumor-associated macrophages (TAMs) in tumor progression.

## 2. Properties of Polarized M1 and M2 ****Macrophages

Macrophages can exert different properties when polarized with distinct inducers. Differential cytokine production is a key feature of polarized macrophages. When stimulated with IFN-*γ*, M1 macrophages secrete high levels of IL-12 and IL-23 but low levels of IL-10 [3, 12–14]. In contrast, M2 macrophages express high levels of IL-10 but low levels of IL-12 and IL-23 [14, 15]. Because of their different cytokine profiles, these polarized macrophages have distinct functions. For example, the IL-12 produced by M1 macrophages can promote the differentiation of Th1 cells, which can improve antigen phagocytosis [12, 16]. IL-23, which is also secreted by M1 macrophages, is associated with the development and expansion of Th17 cells, which can secrete high levels of IL-17 and contribute to inflammatory autoimmune pathologies [17, 18]. In addition, the IL-10 expressed by M2 macrophages can promote the production of IL-4 and IL-13 by Th2 cells [19]. IL-4 is a major promotor of wound healing because it can activate arginase, which contributes to the production of the extracellular matrix. The differential metabolism of L-arginine provides a means of distinguishing the two macrophage activation states. M1 macrophages upregulate iNOS to catabolize L-arginine to nitric oxide (NO) and citrulline, but M2 macrophages induce arginase 1, which metabolizes arginine to ornithine and polyamines, which are precursors necessary for collagen synthesis and cellular proliferation [20]. 

Differentially polarized macrophages can also express different chemokines. For instance, LPS and IFN-*γ* induce macrophages to express chemokine (C–X–C motif) ligand 9 (CXCL9), CXCL10, and CXCL5 through the activation of the transcription factor IFN regulatory factor-3 (IRF-3), which results in IFN-*β* expression and subsequent STAT1 (signal transducer and activator of transcription 1) activation. These proinflammatory chemokines can promote the recruitment of Th1, Tc1, and NK cells, which can improve their capacity for intracellular pathogen killing [10]. In contrast, M2 macrophages inhibit CXCL9, CXCL10, and CXCL5 by downregulating NF-*κ*B and STAT1 [21, 22]. M2a macrophages induced by IL-4 and IL-13 promote the expression of Chemokine (C–C motif) ligand 24 (CCL24), CCL17, and CCL22. These chemokines can specifically combine with Chemokine (C–C motif) receptor 3 (CCR3) and CCR4, which accelerate the recruitment of eosinophils, basophils, and Th2 cells, to lead to a type II response. M2b cells always secrete CCL1, which combines with CCR1 to promote the infiltration of eosinophils, Th2, and regulatory T cells. These cells exert immune regulation and drive the Th2 response. Finally, in M2c macrophages, IL-10 induces CXCL13, CCL16, and CCL18, which can combine with CXCR5, CCR1, and CCR8 to promote the accumulation of eosinophils and naïve T cells, which play a prominent role in suppressing immune responses and promoting tissue remodeling [10]. In considering the above information, we found that the role of chemokines expressed by different macrophages is in accordance with the cytokines they express. The M1-derived chemokines are important for killing intercellular pathogens, whereas the M2-derived chemokines promote the recruitment of the leukocytes involved in tissue repair and remodeling. 

Heterogeneity and plasticity are important features of macrophages. Under different stimuli, macrophages can polarize into different phenotypes. However, these phenotypes are not stable. Several in vivo studies have demonstrated that the phenotype of an activated macrophage population can change over time. For example, during tumor progression, the macrophage phenotype changes from classically activated to alternatively activated [23]. In contrast, the macrophage phenotype changes from M2 to M1 in obesity [24]. However, clarification on whether this phenotypic alteration is the result of a dedifferentiation of the original macrophages back to the resting state or the migration of a new population of macrophages into the tissue site that replace the original cells is still needed. In vitro investigations have clearly shown that polarized macrophages (M1 or M2) change their expression profile according to changes in stimuli [2]. Therefore, macrophages could repolarize in response to changes in the local microenvironment, allowing them to shape the local inflammatory milieu to adapt to outside stimuli. High plasticity is an important characteristic of macrophages and contributes to the development of certain disorders. 

As we mentioned above, macrophages in different microenvironments play different roles. Next, we will discuss the role of TAMs in tumor progression.

## 3. The Role of TAMs in Tumor Progression

A tumor, as defined by Wills, is “an abnormal mass of tissue, the growth of which is uncoordinated with that of the normal tissues and persists after the cessation of the stimuli which evoked the change.” Tumors are composed of proliferating tumor cells and stromal cells, including endothelial cells, inflammatory cells, and fibroblasts [25]. In the 1970s, it was found that TAMs, as the predominant leukocyte, play a key role in tumor growth [26]. 

The role of TAMs in tumors is still controversial. It has been reported that in colorectal tumors TAMs are proinflammatory, and play an antitumor role, which leads to a good prognosis [27, 28]. One possible reason is that the M1 TAMs promote colon tumor cell expressing galcetin-3 which further induce more TAMs infiltration and lead to an amplification immune response to destruct tumor cells [28]. On the other hand, TAMs express a series of proinflammatory cytokines such as IFN-*γ*, IL-1, and IL-6, which activate type-1 T-cell associated with antitumor immune responses [27]. However, in most tumors such as breast, prostate, ovarian, cervical, lung carcinoma, and cutaneous melanoma, TAMs are considered to be antiinflammatory and correlated with a poor prognosis. Epidemiological studies have suggested that a macrophage-rich microenvironment will promote an aggressive tumor with a high metastatic potential [29]. Therefore, many scholars have further studied the function of TAMs in tumorigenesis. In the present study we will focus on how the anti-inflammatory TAMs influence the progression of tumors. 

**Figure 2:**
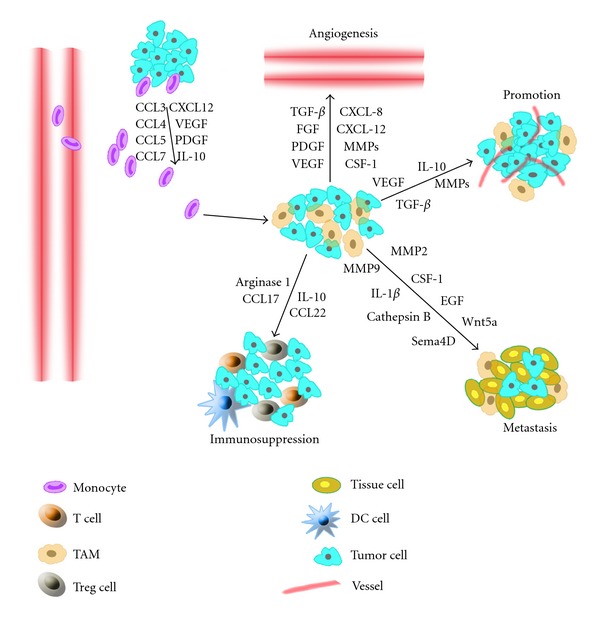
TAM functions in tumor progression. Tumor cells and stromal cells, which produce a series of chemokines and growth factors, induce monocytes to differentiate into macrophages. In the tumor, most macrophages are M2-like, and they express some cytokines, chemokines, and proteases, which promote tumor angiogenesis, metastasis, and immunosuppression.

TAMs exhibit an M2-like phenotype because they express a series of markers, such as CD163, the Fc fragment of IgG, C-type lectin domains, and heat shock proteins [30–32]. On the other hand, the tumor microenvironment includes a number of chemoattractants, such as IL-4, IL-13, TGF-*β*, and IL-10, all of which lead to the adoption of an M2 phenotype [33]. TAMs orchestrate various aspects of cancer, such as tumor progression, angiogenesis, tumor growth, actual metastasis, immunosuppression, matrix deposition, and remodeling (Figure 2). 

### 3.1. Monocyte Recruitment

TAMs are differentiated from monocytes by a number of chemoattractants that are produced by tumor cells and stromal cells. For instance, tumor-derived chemokine CCL2, formerly known as monocyte chemotactic protein (MCP), is critical for the recruitment of macrophages [34, 35]. CCL2 is produced by tumor cells, fibroblasts, and macrophages, and high CCL2 levels are correlated with increased numbers of TAMs and a poor prognosis [36]. Other chemokines, such as CCL3, CCL4, CCL5, CCL7, CCL8, CXCL12, and cytokines, including vascular endothelial growth factor (VEGF), platelet-derived growth factor (PDGF), and IL-10, are also reported to promote macrophage recruitment [14, 37–39]. In addition, another group of monocyte chemoattractants, the alarmins, have been reported to promote the recruitment of monocytes and other myeloid cells [40]. For example, the high mobility group box protein 1 (HMGB1), which is one of the molecules released by dying tumor cells, is found in the necrotic areas where TAMs preferentially reside. Other alarmins, such as S100A8, S100A9, serum amyloid A3 (SAA3), and fibronectin, have also been reported to attract CD11b^+^ myeloid cells [41].

### 3.2. TAMs and Angiogenesis

Tumors do not grow beyond 2-3 mm³ and cannot metastasize unless they are vascularized [42]. It is well known that the growth and spread of malignant tumors requires angiogenesis, the process by which new blood vessels sprout from the existing vasculature. Accumulating evidence indicates that TAMs play an important role in regulating angiogenesis. Bingle and his colleagues demonstrated that TAMs present within a solid tumor significantly contribute to the initiation of angiogenesis. In the absence of TAMs, the tumor cells produce the necessary stimuli to initiate tumor angiogenesis, but the initiation is delayed [43]. More recently, Zeisberger et al. found that depleting TAMs with clodronate encapsulated in liposomes (clodrolip) could reduce blood vessel density in the tumor tissue [44]. These results validate the idea that TAMs present in the tumor microenvironment promote angiogenesis in tumors.

How do the TAMs regulate angiogenesis? It has been reported that TAMs are able to modulate and induce neovascularization and support functions. When TAMs are activated, they can express a broad repertoire of substances (including growth factors, cytokines, proteases, and chemokines) to promote angiogenesis. For instance, TAMs release growth factors such as VEGF, PDGF, transforming growth factor *β* (TGF-*β*), and a member of the FGF family, which can promote angiogenesis in many tumors, such as gliomas, squamous cell carcinomas of the esophagus, and breast, bladder, and prostate carcinomas [14, 36, 45]. In addition, Aharinejad et al. found that the overexpression of colony-stimulatingfactor 1 (CSF-1) can enhance the recruitment of TAMs, which accelerates tumor development and malignant progression in the mammary epithelium of MMTV-PyMT mice [46]. Lin and colleagues found that, when inhibiting the expression of CSF-1 or its receptor with short-interfering RNA (siRNA) in mice model, macrophage infiltration and vascularity are decreased compared to their CSF-1 counterparts [47, 48]. Moreover, TAM-derived proteases, such as matrix metalloproteases (MMP-1, MMP-2, MMP-3, MMP-9, and MMP-12), plasmin, and urokinase plasminogen are also beneficial to angiogenesis. MMP-9 is one of the most important proteases that degrade the extracellular matrix (ECM) and further release other growth factors to stimulate angiogenesis [49, 50]. MMP-2 expression is also increased in several tumors, which is correlated with the nodal status and tumor stages [51].

TAMs have been found to accumulate in hypoxic regions of tumors, which are characterized by low-oxygen tension. As TAMs adapt to the hypoxic microenvironment, they can express more proangiogenic genes, such as VEGF, pFGF, CXCL8, and glycolytic enzymes, whose transcription is controlled by the transcription factors HIF-1 and HIF-2 [42]. In addition, it has been reported that the HIF-1-dependent chemokine CXCL-12 acts as a potent chemoattractant that promotes endothelial cell infiltration when specifically combined with its sole receptor, CXCR4 [52]. 

### 3.3. TAMs and Lymphangiogenesis

Lymphangiogenesis is the initial step in the generalized spread of tumor cells, which predicts a poor clinical prognosis. TAMs promote the lymphangiogenesis mediated by VEGF-C and VEGF-D via VEGFR3 [53]. It has been reported that VEGF-C and VEGF-D are produced not only by tumor cells but also by TAMs. In human cervical cancer, the VEGF-C released by TAMs plays a novel role in peritumoral lymphangiogenesis and the subsequent formation of lymphatic metastases [54]. However, in bladder cancer, VEGF-C expression was positively associated with both lymphangiogenesis and angiogenesis, while VEGF-D was associated only with lymphangiogenesis [55]. In addition, TAMs can express lymphatic endothelial growth factors to promote lymphangiogenesis [54, 56].

Recently, Maruyama and colleagues found that CD11b^+^ macrophages physically contribute to lymphangiogenesis under pathological conditions and that bone marrow-derived CD11b^+^ macrophages express lymphatic endothelial markers, such as LYVE-1 and Prox-1, under inflamed conditions in the corneal stroma of mice [57]. These findings suggest that macrophages induce lymphangiogenesis in two different ways, either by transdifferentiating and directly incorporating into the endothelial layer or by stimulating the division of preexisting local lymphatic endothelial cells [58].

### 3.4. TAMs and Tumor Growth

In addition to promoting angiogenesis and lymphangiogenesis, TAMs also play a pivotal role in tumor growth. It has been demonstrated that TAM infiltration is positively correlated with the proliferation of tumor cells in several tumors, such as breast cancer, endometrial cancer, and renal cell cancer [59, 60]. Macrophages cocultured with tumor cells could secrete a series of substances which facilitate tumor cell proliferation [42]. Additionally, macrophage depletion studies have proven that TAMs are essential for tumor growth [61]. 

MMP9, which was mentioned earlier as a primary factor promoting angiogenesis, also plays an important role in tumor growth. The cytokine IL-23 is considered to promote tumor incidence and growth by upregulating MMP9, thereby stimulating inflammatory responses [62, 63]. Moreover, TAMs limit the cytotoxicity of the microenvironment, which helps tumor growth. Because TAMs are M2-like, they can secrete large amounts of IL-10, which can suppress cytotoxic T-cell activity by inhibiting Th1 cells while simultaneously inhibiting NK and lymphokine-activated killer cell cytotoxicity [42]. 

Recently, a notable paper reported that in melanomas, TAM-derived adrenomedullin is involved in angiogenesis and tumor growth. It was found that the adrenomedullin derived from TAMs interacts with its receptors on endothelial cells to promote tumor growth via a paracrine loop through the activation of the eNOS signaling pathway similar to the angiogenesis cytokine VEGF [64]. On the other hand, TAM-derived adrenomedullin can influence macrophages themselves in an autocrine manner. The inhibition of adrenomedullin receptors on TAMs impairs angiogenesis and tumor growth [65, 66]. 

### 3.5. TAMs and Tumor Metastasis

Tumor metastasis is an important marker in determining the severity of cancer. Tumor cells metastasize through the blood and lymphatic vessels, which leads to the formation of ectopic tumors. These tumors present a great therapeutic challenge and result in a poor prognosis [67]. More than 20 years ago, with mouse models, Gorelik et al. found that TAMs promote tumor metastasis. After intravenous injection of murine tumor cells, the macrophage population increased during the formation of lung tumor nodules [68]. Subsequently, Wyckoff and colleagues demonstrated a synergistic relationship between breast cancer cells and TAMs in cell migration [67].

Coffelt et al. found that both TAM and tumor cells migrated frequently when they were in close proximity by multiphoton microscopy. Further study revealed that the epidermal growth factor (EGF) released by TAMs interacted with the CSF-1 released by tumor cells to promote the migration of the tumor cells [41]. Accumulating studies have verified that the malignant cells always move next to the TAMs, which appear to help malignant cells during intravasation [69]. Pawelek and Chakraborty even proposed that when cancer cells fuse with migratory bone marrow-derived cells, they provide the driving force behind the dissemination process [70]. 

On the other hand, TAMs influence the microenvironment, which can also promote tumor cell invasion. Hagemann and colleagues demonstrated that coculturing TAMs with tumor cells can promote the expression of MMPs, especially MMP2 and MMP9, in TNF-*α*-dependent manner [71]. Both MMP2 and MMP9 help degrade the proteins in the extracellular matrix to promote metastasis [42]. In addition, Seth et al. showed that MMP7 could also promote tumor metastasis through converting the receptor activator of nuclear factor *κ*B ligand (RANKL) [72, 73]. Other macrophage-derived molecules, such as IL-1*β*, cathepsin B, Wnt5a, and semaphorin 4D (Sema4D), have also been reported to promote tumor metastasis [41].

Recently, it has been reported that macrophage-derived microRNA (miRNA) also regulated tumor invasion. Yang et al. found that oxesomes containing miR-233 shuttle between macrophages and breast tumor cells. miR-233-regulating tumor invasion is considered to work through the mef2c-*β*-catinen pathway [74]. This research indicated that cell-to-cell interaction is not only restricted in protein but also provides us a new research direction in future.

### 3.6. TAMs and Immunosuppression

Tumor immunosuppression is a well-established mechanism for the regulation of tumor growth. Several studies have reported that TAM-derived cytokines and proteases, such as TGF-*β*, IL-10, and arginase 1, make a significant contribution to immunosuppression [75–77]. For instance, TGF-*β* has a crucial immunosuppressive role in both the innate and the adaptive arms of the immune response. In the innate immune response, TGF-*β* promotes tumor-associated macrophage polarization to an M2-versus-M1 phenotype, which further promotes TGF-*β* production and deepens immunosuppression [78]. TGF-*β* also inhibits the cytolytic activity of natural killer (NK) cells expressing the activating receptor NKG2D, further resulting in a poor antitumor response [79, 80]. In addition, TGF-*β* decreases dendritic cells (DCs) migration and increases apoptosis, which decreases antigen presentation and dampens the adaptive immune response [81, 82]. In the adaptive immune response, TGF-*β* promotes CD4^+^ T cells differentiation into Th2 cells rather than Th1 cells, which promotes a less efficient antitumor immune response [83]. TGF-*β* also inhibits the CD8^+^ T cells antitumor activity by suppressing the expression of several cytolytic genes, including the genes encoding granzyme A, granzyme B, IFN-*γ*, and FAS ligand [79, 84]. Furthermore, TGF-*β* promotes tumor growth by the maintenance of Treg cell differentiation, which inhibits the antitumor response [79].

IL-10, an important cytokine in the tumor microenvironment, is expressed by TAMs, CD8^+^ T-cells, and tumor cells. IL-10 is commonly regarded as an anti-inflammatory, immunosuppressive cytokine that favors tumor escape from immune surveillance. TAM-derived IL-10 acting in an autocrine circuit suppresses the expression of IL-12, a potential antitumor cytokine [85]. Several studies have reported that TAM-derived IL-10 prevents the maturation of DCs in situ but increases the differentiation of macrophages, which decreases antigen presentation [76, 86]. IL-10 can also inhibit the release of the cytotoxic cytokine IFN-*γ*, which is the main factor that stimulates naïve T-cell differentiation, to promote immune evasion [87]. Furthermore, it has been reported that IL-10 decreases the ability of epidermal APCs to present tumor-associated antigens for the induction of antitumor immune responses in a spindle cell tumor system [88]. However, not all agree with that IL-10 leads to immunosuppression. Some articles reported that IL-10 possesses some immunostimulating properties, which play important roles in antitumor response [89–91]. For example, in NSCLS stage I, it is found that the more infiltrating CD8^+^/IL-10^+^ cells there are, the longer the overall survival will be [89]. So the role of IL-10 in tumor microenvironment is still controversial. To make it clear may take a huge forward for tumor therapy. 

 Arginase 1, the molecular marker for M2 macrophages, is highly expressed in tumors. In recent years, it has been demonstrated that arginase 1, which primarily metabolizes L-arginine into polyamine and proline, causes dysregulation of the T cell receptor (TCR) signal and subsequently induces CD8^+^ T cell unresponsiveness [77, 92]. In addition, several studies have reported that arginase 1 activation is associated with H_2_O_2_ production by myeloid-derived suppressor cells (MDSCs), which present class I-restricted epitopes directly to CD8^+^ T cells and inhibit their release of IFN-*γ* through the contact-dependent production of H_2_O_2_ [93]. However, the concrete mechanism underlying the H_2_O_2_ generation following arginase 1 activation is not clear and may be linked to the synchronous activation of a different NOS isoform [92].

Finally, several studies have found that chemokines also play an important role in immunosuppression. Chemokines, such as CCL17 and CCL22, can prevent the infiltration of cytotoxic T-cells but promote that of Treg and Th2 cells [38, 94]. TAM-derived CCL18 has the ability to recruit naïve T-cells, which induces T-cell anergy [14]. CCL-2 and CCL-5, which were mentioned previously as chemoattractants of monocytes to tumors, induce suppression of T-cell responses [76]. Further studies have shown that TAM-induced immunosuppression is correlated with the activation of transcription factors, such as STAT3, STAT6, and NF-*κ*B, but the specific mechanism still needs to be explored [41].

### 3.7. Interaction between TAM and Cancer Stem Cells (CSCs)

Over the past 5 to 10 years, it has been found that a specific subpopulation of tumor cells has distinct stem cell properties in tumors. These cells are defined as cancer initiating cells or cancer stem cells (CSCs). A CSC has the ability to initiate tumorigenesis by undergoing self-renewal and differentiation [95, 96]. However, stromal cells, such as fibroblasts and immune cells, are also known to play important roles in tumor progression [97]. Therefore, research on the relationship between CSCs and stromal cells has become an exciting area of focus.

TAMs, as the dominant immune cell components, are considered to be closely related to CSCs in position. Several studies have reported that TAMs are always found distributed around CSCs, and the number of infiltrating TAMs has been positively correlated with the histological grade of the malignancy and the number of CSCs found [98, 99]. Furthermore, Yi, et al. found that the production of CSC-derived chemoattractants, including CCL2, CCL5, VEGF-A, and NTS, in glioma tissue was much higher than in adhesive glioma cells (AGCs), which promotes the infiltration of macrophages. However, when a specific antibody to the chemoattractants was used, the migration of the macrophages decreased. These results indicate that CSCs play a more dominant role in recruiting macrophages than AGCs [100]. At approximately the same time as the publication of these results, another paper reported that CSCs in glioma tissue induce macrophage infiltration and polarize the macrophages into an M2 phenotype because the macrophages secreted a large number of cytokines, such as TGF-*β*1, IL-10, and IL-23. In addition, M2 macrophages could induce T-cell anergy and therefore immunosuppression in agreement with what we mentioned previously [101]. Both of these articles indicated that CSCs play a leading role in macrophage infiltration and polarization. 

Recently, other articles have demonstrated that macrophages can also influence the characteristics of CSCs, which promote tumorigenesis and metastasis. For example, Jinushi and colleagues found that CSCs could specifically stimulate TAMs to express the downstream factor milk-fat globule epidermal growth factor VIII (MFG-E8), which has been identified as a growth factor involved in phagocytosis, angiogenesis, and immune tolerance. MFG-E8 induces CSCs to form tumors and develop antitumor drug resistance through the STAT3 and hedgehog signaling pathways [102]. Okuda et al. found another novel phenomenon: highly metastatic breast CSCs upregulate the expression of hyaluronan synthase HAS2, which correlates with tumorigenicity and tumor progression in several cancers. The interaction between CSCs and TAMs through hyaluronan stimulates the secretion of PDGF-BB, which in turn activates stromal cells to secrete the FGF7 and FGF9 that stimulate CSC proliferation, self-renewal and metastasis in the bone [103].

All of these studies indicate that macrophages promote CSC proliferation and metastasis. However, the investigation into the interaction between macrophages and CSCs is still at an early stage. More in-depth research requires our joint efforts. 

## 4. Potential Therapies Targeting TAMs

Accumulating studies have demonstrated that the density of TAMs is associated with a poor prognosis, suggesting macrophages as a target for clinical therapy [104–107]. As early as in 1970s, Dolph Adams has raised the point that macrophage mediated tumor cytotoxicity (MTC). It is considered that macrophages can be activated through two steps: The basic step is through the cytokines and other small molecules and the secondary signal is supplied by either antibody or LPS/endotoxin/TLR stimulants. Both of the signals can activate MTC and resist tumor activity [108]. Today it is considered through the following steps such as antimacrophage infiltration, antiangiogenesis, and converting M2 to M1 to resist macrophage-mediated tumor activity.

Several studies reporting the use of “antimacrophage” approaches have primarily focused on counteracting monocyte chemokines and receptors as anticancer targets [10, 14, 36, 42]. In the murine model for breast cancer, macrophages were recruited by the tumor cell-derived chemokine CCL5. After treatment with the receptor antagonist met-CCL5, both the number of infiltrating macrophages and the size of the tumor were significantly reduced [109]. In addition, some studies have shown that Trabectedin, a natural product derived from the marine organism **Ecteinascidia * turbinata*, has a specific cytotoxic effect on human macrophages and TAMs in vitro [110]. Recently, it was reported that pharmacological drugs, such as zoledronic acid combined with sorafenib, enhance antitumor effects by depleting the macrophage population [111]. Other pharmacological drugs, including thalidomide, linomide, pentoxifyline, and genistein, have also been shown to inhibit macrophage infiltration and reduce tumor size [112, 113]. 

As we mentioned earlier, tumors do not grow beyond 2-3 mm^3^ unless they are vascularized, so inhibiting angiogenesis is also a good therapeutic approach. Several studies revealed that anti-VEGF-A with Avastin/bevacizumab or other neutralizing antibodies can both inhibit the infiltration of macrophages and enhance the activity of antiangiogenic therapies by preventing TAMs from secreting additional pro-angiogenic factors [114, 115]. 

As M1 macrophages induce proinflammatory response which protects body from injury, converting the M2 macrophages into M1 is also considered to be a better potential therapy. Several articles have reported that activation of TLRs stimulates M1-polarized macrophage response, which induce the activation of proinflammatory program [116]. In a mouse model, Guiducci et al. found that CpG plus antiinterleukin-10 receptor antibody promptly switched infiltrating macrophages infiltrate from M2 to M1 and triggered innate response debulking large tumors [117]. SHIP1 is a crucial phosphatase in the conversion from macrophage M1 to M2 functions. Therefore, pharmacological modulators of this phosphatase that can promote the infiltration of M1 macrophages and inhibit M2 macrophages, thereby enhancing the antitumor effects of M1 cytotoxicity, are under investigation [14, 118]. 

 In addition, accumulating studies report using macrophages as natural vectors to deliver therapeutic molecules to the neoplastic site [14, 41, 119]. For instance, intratumoral injection of macrophages transfected with an IL-12-expressing recombinant adenoviral vector can enhance the number of CD4^+^ and CD8^+^ cells and reduce tumor growth and metastasis [120]. Moreover, Siveen and Kuttan found that paclitaxel, a plant-derived diterpenoid, can stimulate macrophages to express high levels of NO, TNF-*α*, and IL-1*β*. Through the increased levels of these substances, paclitaxel can enhance tumor cell cytotoxicity and restore IL-12 production by macrophages in tumor-bearing mice [36]. Recently, it was reported that an anti-PD-L1 antibody, which blocks the PD-1/PD-L1 pathway, can improve macrophage-mediated T-cell activation in HCC in vivo and has progressed to a phase I clinical study [121, 122]. Perhaps this antibody will be an effective drug in the future.

## 5. Conclusion

Heterogeneity is one of the most important characteristics of macrophages. In different diseases, macrophages can be polarized into different phenotypes. In most tumors, macrophages are considered to be polarized into the M2 phenotype. TAMs express a series of cytokines, chemokines, and proteases to promote angiogenesis, lymphangiogenesis, tumor growth, metastasis, and immunosuppression. Recently, it has also been reported that TAMs interact with CSCs, which facilitate tumorigenicity, metastasis, and drug resistance. Taken together, these findings indicate that targeting macrophages in the tumor microenvironment may provide more efficacious novel therapies for future tumor management.
